# Guiding Principles for Pragmatic Randomized Trials of Tuberculosis Treatments

**DOI:** 10.1016/S1473-3099(26)00359-2

**Published:** 2026-08-14

**Authors:** Patrick P.J. Phillips, Samuel G. Schumacher, Aparna Anderson, Cara Cassino, Salome Charalambous, Richard E Chaisson, Gavin Churchyard, Francesca Conradie, Christopher Cousins, Gerry Davies, Susan E. Dorman, Hanif Esmail, Katherine Fielding, Carole D. Mitnick, David McNeeley, Payam Nahid, Karen Sanders, Jodie A. Schildkraut, Derek Sloan, Gustavo E. Velásquez, Michael Hoelscher, Andrew Vernon, Christian Lienhardt

**Affiliations:** 1 Center for Tuberculosis, UCSF Institute for Global Health Sciences, University of California, San Francisco, San Francisco, California, USA; 2 World Health Organization, Geneva, Switzerland; 3 Gates Medical Research Institute, Boston, Massachusetts, USA; 4 Stony Point Life Sciences Consulting LLC, New York, USA; 5 The Aurum Institute, Johannesburg, South Africa; 6 Center for Tuberculosis Research, Division of Infectious Diseases, Johns Hopkins University School of Medicine, Baltimore, Maryland, United States of America; 7 Department of International Health, Johns Hopkins Bloomberg School of Public Health, Baltimore, Maryland, United States of America; 8 School of Public Health, University of Witwatersrand, Johannesburg, South Africa; 9 Department of Medicine, Vanderbilt University, Nashville, TN, USA; 10 Faculty of Health Sciences, University of the Witwatersrand, South Africa; 11 Institute for Infection and Immunity, City St George’s, University of London, UK; 12 University of Liverpool, Liverpool, UK; 13 Medical University of South Carolina, South Carolina, USA; 14 UCL Centre for Global Tuberculosis Research and WHO Collaborating Centre for Tuberculosis Research and Innovation, University College London, London, UK; 15 UCL Innovative Clinical Trials Unit, University College London, London, UK; 16 London School of Hygiene and Tropical Medicine, London, UK; 17 Department of Global Health & Social Medicine, Harvard Medical School, Boston, MA, USA; 18 NIH/ NIAID, Bethesda, Maryland, USA; 19 Radboud University Medical Center, Nijmegen, The Netherlands; 20 University of St Andrews, St Andrews, UK; 21 Division of HIV, Infectious Diseases, and Global Medicine, University of California, San Francisco, San Francisco, California, USA; 22 Institute of Infectious Diseases and Tropical Medicine, LMU University Hospital, LMU Munich, Munich, Germany; 23 Unit Global Health, Helmholtz Zentrum München, German Research Center for Environmental Health (HMGU), Neuerburg, Germany; 24 German Centre for Infection Research, Partner Site Munich, 80799 Munich, Germany; 25 Fraunhofer Institute for Translational Medicine and Pharmacology ITMP, Immunology, Infection and Pandemic Research, Türkenstraße 87, 80799 Munich, Germany; 26 French Institute for Research on Sustainable Development (IRD), University of Montpellier, Montpellier, France; 27 FAST-TB Program, CRDF Global, Arlington, VA, USA

## Abstract

Tuberculosis (TB) remains a major global health challenge, despite recent advances in drug and regimen development. Pragmatic randomized trials are needed to evaluate the effectiveness, safety, and tolerability of TB treatments under routine care conditions to appropriately inform practice guidelines and facilitate uptake. We propose guiding principles for pragmatic TB treatment trials. Using the PRECIS-2 framework, we address eligibility, recruitment, setting, organization of care, adherence support, outcomes, follow-up, primary analysis, data collection, and monitoring. Pragmatic trials should maximize generalizability through broad inclusion criteria, integration within routine health systems, and flexible delivery approaches while maintaining appropriate standards for participant safety, laboratory quality, and data reliability. We discuss considerations for individually randomized and cluster-randomized designs, outcome definitions, risk-proportionate monitoring, and streamlined data collection. Adoption of these principles will improve the generation of real-world evidence and facilitate global implementation of effective TB treatments.

## 1 Introduction

Tuberculosis (TB) continues to be the world’s deadliest infectious disease with 10.7 million new cases and 1.23 million deaths in 2024^1^. New treatment regimens are needed to bring the epidemic under control. Despite regulatory approval of three new drugs for the treatment of TB in the past 15 years^1^, weak (i.e., “conditional”) World Health Organization (WHO) recommendations is a key factor impeding uptake of these innovations in high TB-burden countries. Recent phase 3 trials of new regimens for the treatment of TB have been more explanatory in design, which has posed challenges for experts developing global and national treatment guidelines in interpreting the results for a real-world context and led to a disconnect between promising trial results and how the intervention is subsequently implemented by national TB programs^2^. An example is the 4-month rifapentine-moxifloxacin regimen, which was shown to have non-inferior efficacy and comparable safety in a large phase 3 trial with an explanatory design^3^. While this regimen has been recommended by the US Centers for Disease Control and Prevention (CDC)^4^, US- and European-based professional societies^5^, and the WHO^6^, its global uptake has been limited: only 4154 people in 15 countries started treatment with this regimen in 2024, less than 0.1% of the approximately 8 million new cases diagnosed and started on treatment.^1^ There are reports of reduced tolerability in settings where the regimen has been implemented^7,8^, findings that might have been identified in a more pragmatic trial.

A randomized controlled trial is ***explanatory*** if the objective is to evaluate the intervention under ideal conditions; it is ***pragmatic*** if the objective is to evaluate the intervention under conditions that are close to usual practice. Different features in the design and conduct of a trial make it more pragmatic or more explanatory. The explanatory-pragmatic distinction is a continuum, not a dichotomy^9^. Most phase 3 TB trials are explanatory and are characterized by more selective eligibility criteria, standardized interventions and comparators, strict monitoring, and intensive follow-up. New regimens, however, may have certain practical advantages, including shorter duration, improved tolerability, or increased pharmacokinetic forgiveness (the ability of a drug to maintain therapeutic efficacy despite poor adherence)^11,12^. Such benefits could improve programmatic effectiveness by, for example, improving adherence and reducing loss to follow-up in real-world settings, which may not be measurable in a more explanatory trial. These aspects are better evaluated in pragmatic trials whereby promising TB treatment regimens are tested in real-world conditions utilizing the gold standard of randomization^10^, providing further evidence in larger study populations, and thus facilitating their uptake by national programs.

KITT (Knowledge Integration for TB Treatment Development), one of four tracks in the FAST-TB (Facilitating Accelerated Science and Translation for TB Regimen Development) program^11^ (https://fasttb.org/), is a collaboration of TB clinical trial experts with representation from funding agencies, academic research institutes, advocates, and industry. Leveraging the expertise from KITT and best practice from the literature, the objective of this review is to provide a sound scientific framework for pragmatic TB trials.

### 1.1 Structure of review

We have used the nine domains of the PRECIS-2 tool^9^ to organize our recommendations for TB pragmatic trials: eligibility, setting, recruitment, organization, flexibility in delivery, flexibility in adherence, primary outcome, follow-up, and primary analysis. We have included three additional topics: the unit of randomization (addressed first), data collection, and trial monitoring (each addressed last).

### 1.2 Methodology

All members of the KITT initiative (https://fasttb.org/kitt/) were invited to join the working group that developed these guiding principles. We included additional experts with experience in trials of rifamycin-containing regimens or who were actively developing pragmatic trials evaluating these regimens. We conducted a series of webinars among co-authors, during which different experts presented their perspectives alongside published literature on various aspects of the design and conduct of pragmatic TB trials, and led ensuing discussion.

#### 1.2.1 Search Strategy

In preparation for the webinars and in the development of the manuscript, PubMed searches were conducted, along with reviews of appropriate textbooks and TB conference proceedings. The search strategy was specific to each domain under consideration, such as approaches to eligibility criteria, guidance on advantages and disadvantages of cluster-and individually-randomized pragmatic trials, and appropriate monitoring strategies for pragmatic trials.

### 1.3 Criteria for regimens for a TB pragmatic trial

Pragmatic trials are usually appropriate for treatment regimens with strong evidence supporting their efficacy and safety, typically from large randomized phase 3 trials. Reduced protocol-driven monitoring in pragmatic designs demands a high level of confidence in the benefit-risk profile. Thus, pragmatic trials can be described as testing authorized medicinal products either according to the marketing authorization or outside of it, but supported by published evidence, recognized guidance, or established medical practice (categories ‘A’ and ‘Ba’ in the OECD clinical trial risk categorization^12^). Currently, we consider that oral rifamycin-based regimens lasting between four and six months meet these criteria (although shorter durations might be considered^13^). Table 1 summarizes regimens that could be suitable for a pragmatic trial evaluating first-line drug-sensitive TB treatment based on current evidence. Many regimens include drugs for which drug-resistance testing is challenging or not easily interpretable, which introduces additional variability in results. In any case, the principles outlined here can easily be applied to other regimens as more data become available. These principles would be suited for a non-regimen treatment-related intervention or strategy, or other regimens that would meet the OECD criteria for specific types of TB disease such as bedaquiline-based regimens for drug-resistant TB.

### 1.4 Minimum standards for usual care

Variability in clinical, procedural, and laboratory standards will increase outcome heterogeneity, but this is appropriate, and indeed desirable, if this variability reflects global standard practice. Nevertheless, while the objective of a pragmatic trial is to evaluate the intervention under conditions close to usual care, it is still an ethical obligation of investigators to define minimum standards for usual care that meet global and local norms. Minimum standards for usual care in pragmatic TB trials should align with WHO operational handbooks on TB^14,15^ and include access to rapid molecular diagnostics with drug-susceptibility testing for key drugs, supported by quality-assured laboratory systems with procedures for specimen storage, referral testing, and ongoing quality control. Usual care should also provide WHO-recommended treatment regimens, routine clinical and bacteriological monitoring, management of adverse events and comorbidities, and person-centered adherence support tailored to patient needs and preferences.

## 2 Unit of Randomization

In individually-randomized trials, study participants are randomly assigned to one of the study treatments^16^. In contrast, in cluster-randomized trials, groups or “clusters” of participants are assigned to one of the study interventions^16^. A cluster is usually defined by geographical location, comprising a single health facility or a region containing multiple health facilities. Due to intra-cluster correlation among individuals within the same cluster, individually-randomized trials require fewer participants to achieve the same power, and the statistical analysis is simpler because it does not require adjustment for this correlation. Individually-randomized pragmatic trials are best suited to evaluating interventions delivered directly to individuals, and this is therefore the usual design for randomized trials comparing treatment regimens.

There are several areas, however, where individual randomization can add to the demands of routine TB care. Individually-randomized pragmatic trials require individual informed consent before randomization and the start of treatment, which is usually time-consuming and resource-intensive. In routine care settings, clinicians may find it challenging to treat and manage patients differently within the same setting. In contrast, cluster-randomized trials are easier to implement without disrupting routine care, and interventions can be delivered, for the most part, by routine health staff, which enhances external validity and generalizability. Clusters must be selected such that they are representative of the target patient population, covering a sufficient broad range of settings. Under certain conditions, community-level consent can take the place of individual-level consent for a cluster-randomized trial^17–20^, as was the case for TB studies within the CREATE consortium^21^, provided the individual has already given consent within the health system for the sharing of data for research purposes. Cluster-randomized pragmatic trials permit the evaluation of individual treatment outcomes together with implementation process metrics. However, since enrollment of individuals occurs *after* randomization of clusters has occurred and this knowledge of randomized allocation is known, there is a risk of selection bias. In addition, since there are always fewer units of randomization in a cluster-randomized pragmatic trial than in an individually-randomized pragmatic trial, there is an increased risk of individual-level imbalance between treatment arms.

In summary, an individually-randomized trial is usually preferred to provide more precise and stronger evidence for guidelines with fewer study participants. However, an assessment needs to be made for how disruptive individual randomization will be in routine care for persons with TB in the settings where the trial will be conducted, and steps taken to minimize this disruption as much as possible. If this disruption is severe such that many of the approaches described below to make the trial pragmatic are not possible, a cluster-randomized design may be preferred.

## 3 Eligibility

To the extent medically appropriate, all people newly diagnosed with TB and being started on TB treatment at a participating study site should be included in a pragmatic TB trial. This approach maximizes the generalizability of study results. Depending on characteristics of the regimen under study, it may, however, be necessary to exclude certain priority populations from the trial, as described in Table 2. Any exclusion criterion will make the trial less pragmatic and limit generalizability; it therefore requires strong justification.

Care should be taken to ensure that the study population is representative of the broader population of people treated for TB. Exclusion criteria should not be based on prior treatment, prior episodes of TB, or perceived risk factors for poor adherence or loss to follow-up. Exclusion criteria based on laboratory tests not routinely performed for people receiving standard-of-care treatment for TB should be avoided unless medically necessary and intended to be performed as part of the programmatic implementation of the new regimen.

## 4 Participants and Delivery of care

### 4.1 Setting

Study participants should be recruited and followed within the same setting where they ordinarily receive care. This approach minimizes disruption to participants and reduces additional travel or time commitments outside the usual care. It is also more pragmatic, as participants will be enrolled in the same settings where the trial interventions may ultimately be implemented. Explanatory trials often require a stronger infrastructure and more staff to ensure close compliance with a detailed protocol that will depart from usual practice in many areas. In contrast, a pragmatic trial protocol will be designed to mirror usual practice as much as possible; such trials can be conducted in a broader range of sites and countries.

Adding a clinical trial to routine clinical care will inevitably impose some additional burden on clinic staff, which may have a serious impact on internal validity. This disruption can be minimized by developing research partnerships between investigators and healthcare system representatives and integrating research into the clinical workflow where possible^22^. A pragmatic TB trial requires engagement with the national TB program (NTP) as well as local and provincial health districts that oversee clinical care.

### 4.2 Recruitment

Typically, study participants are asked whether they would be interested in participating in a TB clinical trial when they access care. Any method for engaging potential study participants that would be over and above what would be used in usual care could make a trial less pragmatic, and so the benefits should be carefully weighed. Providing incentives (e.g., financial compensation) and enablers (e.g., travel vouchers) that are not components of usual care and are not planned as part of any future programmatic rollout of the new treatment will make a trial less pragmatic. However, if participation in the trial requires additional visits or procedures beyond standard practice, participants may need to be reimbursed for related travel, time, or other reasonable expenses, in accordance with ethical guidelines and local regulations.

### 4.3 Organization and flexibility in delivery of care

In pragmatic TB trials, the organization of care delivery in the intervention arm should closely mirror that of routine TB services, with minimal deviation from standard practice. This includes drug procurement, storage, dispensing, and prescribing. Clinical management should be conducted within the existing health system by usual care providers, using standard resources and workflows. This embedded design minimizes the need for specially trained or certified trial staff. Activities that overlap with routine TB management (e.g., sputum collection, clinical assessment) are performed as part of normal care and are not classified as study procedures. This organization ensures that trial operations are integrated into the established TB care infrastructure. Only procedures that are distinct from routine care, such as randomization, additional specimen collection, specific adverse event reporting beyond standard practice, deviations from normal prescribing practice, or dedicated trial data entry, require staff trained in Good Clinical Practice (GCP) and study-specific procedures, although this will depend on the regulatory framework under which the trial is conducted. If the intervention regimen includes drugs not part of routine TB treatment, specific guidance would be needed on medication administration (e.g., with or without food), advice and restrictions on co-interventions, and on potential side effects. Furthermore, it may be appropriate to include a central clinical advisory committee that can be available for consultation in case of unexpected safety events or uncertainty regarding treatment decisions in the event of possible treatment failure or relapse. Such a central clinical committee would be a departure from usual care and thus make the trial less pragmatic, but may be considered appropriate to protect safety of study participants.

## 5 Flexibility in adherence

Approaches to support patient adherence to treatment vary widely. The 2025 WHO consolidated guidelines on tuberculosis^23^ state that “*treatment support needs to be provided in the context of people-centred care and should be based on the individual patient’s needs, acceptability and preferences*.”^23^; this can be achieved through several care and support interventions, including health education and counseling, digital medication monitors, material and psychological support, community- or home-based treatment support, and video-supported treatment. The guidelines recognize that these are not mutually exclusive and should be offered to patients as part of a package.

Many TB clinical trials that are more explanatory in design have included the requirement that trial participants are directly observed daily as they take their medication, either by someone from the clinic or from the community^3,24^ In contrast, to promote generalizability, a pragmatic trial should allow flexibility in adherence support and therefore not include additional measures beyond usual care, provided usual care is broadly consistent with best practice and WHO guidelines, although this can increase heterogeneity of trial results and may require an increase in sample size. An exception would be when the regimen is intended to be implemented alongside a specific adherence-promoting tool.

Separately from adherence *support*, the *measurement* of treatment adherence has had an important role in understanding the results of explanatory trials^25^. This, however, is not a priority in a pragmatic trial and should be included only if it does not change the approach to routine treatment support.

## 6 Outcome definitions

### 6.1 Primary outcomes

Key outcomes rated as critical by the WHO guidelines development group (GDG) members in the evidence review process include: sustained cure, death, TB treatment failure & recurrence, loss to follow-up, severe and serious adverse events, acquisition of drug resistance, and intolerability^26^. Pragmatic trials should have a single primary outcome; other important outcomes can be included as secondary^27^.

The primary outcome of a pragmatic trial should measure directly how a patient ‘feels, functions, or survives’^28^. Follow-up after the end of treatment is necessary to detect any recurrence of disease. As around 70% of recurrences occur within six months of treatment completion in TB trials ^29,30^ and given that the current standard of care regimen is of six months’ duration, a total of 12 months from treatment initiation should be considered the absolute minimum follow-up duration for a TB pragmatic trial. In line with this, recent TB trials have used a twelve-month post-treatment initiation outcome as primary^3^, with consistent results in the secondary eighteen-month outcome^31^. While too short a follow-up can limit confidence that sustained cure has been achieved, too long a follow-up can lead to high rates of losses to follow-up, exogenous reinfection with a new TB strain, and missing data. As practical guidance, and since the objective of a pragmatic trial is to generate evidence to convince a broad range of stakeholders, we recommend that the primary outcome of a TB pragmatic trial be sustained cure at a fixed timepoint of two years after treatment initiation, sustained cure being defined as cure at the end of treatment associated with disease-free survival at two years without need for additional treatment beyond the allocated regimen.

Determination of disease-free survival at two years after treatment initiation requires an assessment at the end of follow-up that (i) the study participant is free from signs and symptoms of TB disease (without another definite diagnosis) and (ii) bacteriological evidence of absence of disease. The first criterion meets the need to have an outcome that measures how a patient ‘feels, functions, or survives’, but the second criterion is necessary to rule out asymptomatic disease. Bacteriological evidence would ideally be a negative sputum culture result. However, recognizing potential resource implications, strategies such as performing a nucleic acid amplification test (NAAT) on sputum followed by sputum culture only for participants with a positive NAAT test could be considered. In addition, if the trial includes individuals with extrapulmonary TB, clinical and/or radiological evidence may need to suffice for the determination of absence of disease. Linkage with national TB and death registries, where available, can support triangulation of outcome data.

#### 6.1.1 Follow-up duration for shorter regimens

Although the most common standard of care treatment for TB is six months, newer regimens being evaluated in clinical trials are often shorter, even as short as two months^32^. A perennial question is whether the duration of follow-up from treatment initiation in a TB clinical trial should be the same for all regimens, or whether it should be shorter for shorter regimens to ensure consistent post-treatment follow-up across regimens. The former approach is favored by most authors and recommended in consensus guidance for TB trials^26,33^ as it preserves randomized comparability, avoids bias in favor of shorter regimens, and is consistent with the ‘time zero’ rules in the context of a causal inference framework^34^. The latter approach is advocated by some because it avoids bias in favor of longer regimens and strikes a balance between a manageable burden of follow-up, capturing most recurrences, and minimizing losses to follow-up and exogenous reinfections.

### 6.2 Secondary efficacy outcomes

Aside from the primary outcome, several outcomes are typically of interest to TB guidelines development groups.

- *All-cause mortality during treatment and follow-up*, together with the primary cause of death where possible.
- *TB treatment failure*, defined as the absence of cure at the end of treatment that requires an extension of treatment (based on any combination of bacteriological, radiological, and/or clinical factors).
- *Recurrence*, defined as the requirement for additional treatment (based on any combination of bacteriological, radiological and/or clinical factors) after the end of treatment.
- *Lost to follow-up*, defined as inadequate evidence of disease-free survival at the end of follow-up when there is no alternative evidence of death, failure, or recurrence.
- *Acquisition of drug resistance*, defined as treatment failure or recurrence, where the TB bacterial strain exhibits new resistance to drugs that was not present in the strain that infected the study participant at the start of treatment. Determination of this outcome requires resistance testing of TB isolates both at baseline and at the time of treatment failure or recurrence. In many settings, this is part of local practice. Where not routinely performed, we propose that this nevertheless be performed in the context of a pragmatic trial as it does not interfere with the pragmatic delivery of care within the trial and has important implications for future regimen use at both the individual and community level.

### 6.3 Safety and tolerability

In pragmatic trials, safety monitoring must balance the ethical obligations of investigators to participants alongside the intent to study the treatments under “real-world” conditions. We recommend using a risk-proportionate approach that integrates with routine clinical workflows, yet is based on the known safety profile of the drugs used in the trial and aligns with Good Clinical Practice (GCP) and regulatory requirements. For regimens including drugs with a well-understood safety profile, clinical monitoring included in the local standard of care could be considered the primary mechanism for safety management. Early consultation with stakeholders, including community members and ethics boards, is critical for the selection of a clinical safety monitoring strategy that both prioritizes the welfare of participants and addresses gaps in knowledge about the safety of the treatment under routine care conditions.

It is worth pointing out that, depending on the specific research question, data on safety outcomes may still be collected even if these events are expected or part of routine clinical management. In addition, because pragmatic trials often have broader eligibility than explanatory trials, participants may have more comorbidities and concomitant medications, leading to a higher volume of adverse events and decreased certainty about the relatedness of an event to trial participation. A key principle is that ‘real world conditions’ are best matched by having a simple mechanism for reporting safety outcomes in the database and to the sponsor (and other parties such as regulators and ethics committees if required) rather than by limiting the reporting.

Tolerability can be measured through the proposed proxies of treatment interruptions and non-completion of treatment^35,36^. It may be desirable to also capture treatment acceptability and patient-reported outcomes directly^37^, although the extent to which measuring those outcomes impacts trial pragmatism must be carefully considered.

## 7 Follow-up schedule

The intensity and duration of follow-up of participants is informed by the choice of primary and secondary outcomes. For the primary outcome, as described above, it will be necessary to capture data for two years after treatment initiation to properly assess the effectiveness of a regimen in achieving sustained cure.

**Figure 1:**
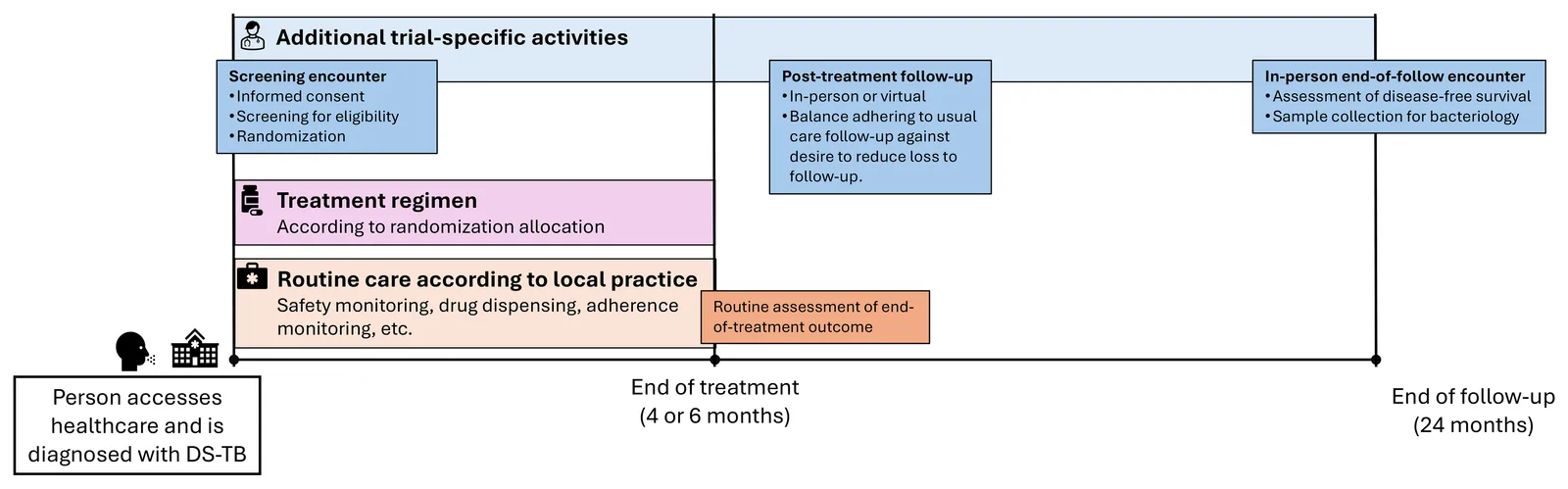
Illustration of participant flow through the TB pragmatic trial with schedule of events indicating which are routine and which are trial-specific.

In general, the schedule of tests and measurements at each visit *during treatment* should follow local practice (see Figure 1). This may include in-person clinic visits, virtual phone-based encounters, or community/home visits. Depending on the intervention regimen, there may be additional safety monitoring for expected toxicities, which should then be envisioned as a routine part of implementation of the regimen.

Post-treatment follow-up is not routine in many programmatic settings but is required for effectiveness assessment in the context of a TB pragmatic trial and, therefore, will necessarily depart from pragmatic trial principles. When not routine, infrequent follow-up can help maximize trial pragmatism; however, outcome ascertainment may be biased if infrequent follow-up inadvertently lead to high rates of loss to follow-up. This must be addressed in the statistical analysis as described below. Special care needs to be taken to conduct follow-up and define loss to follow-up consistently across arms. Determination of disease-free survival after treatment initiation requires that the participant is found free from signs and symptoms of TB disease and that there is bacteriological evidence of absence of disease. Accordingly, at a minimum, a single in-person end-of-follow-up encounter is necessary to capture these data. Such an in-person visit could take place in the clinic, the community, or at home, depending on the needs for sample collection and the participant’s preference.

## 8 Primary Analysis

The primary analysis of a TB pragmatic trial should include all randomized participants in the group to which they were randomized, irrespective of whether treatment was started, changed, or completed. In a cluster-randomized trial, this would include all participants within the defined cluster during the period of observation.

### 8.1 Estimands and the handling of intercurrent events

The ICH E9(R1) addendum introduces the requirement that a clinical trial should define the primary estimand of interest in addition to the primary outcome. In this context, an estimand is “a precise description of the treatment effect reflecting the clinical question posed by the trial objective.”^38^ Clear specification of the estimand requires five attributes: the treatment conditions being compared, the patient population targeted by the scientific question, the outcome or endpoint of interest, how intercurrent events will be handled in the analysis, and the statistical summary measure.^39^ Intercurrent events are defined as events that occur after randomization and either preclude observation of the final endpoint or affect its interpretation. Common intercurrent events in more explanatory TB clinical trials include treatment changes, events leading to treatment withdrawal or loss to follow-up, a restart or extension of treatment, death unrelated to TB disease, isolated positive cultures that may not indicate recurrence, and exogenous reinfection.

For a pragmatic TB trial, any death should be classified as absence of sustained cure as should any diagnosis of treatment failure or recurrence of disease for which a new treatment course or treatment extension is indicated (the composite strategy^38^). Unlike more explanatory TB trials, where whole-genome sequencing is often used to distinguish exogenous reinfection from endogenous relapse, these should be grouped together as disease recurrence in the primary estimand in a pragmatic trial, since both are undesirable outcomes from a patient perspective and whole-genome sequencing is not routine in many programmatic settings. Treatment changes for adverse events, intolerability, or accounting for inadequate adherence should not be classified as absence of *sustained cure* in a pragmatic trial but be considered as part of a pragmatic implementation of the regimen (the treatment policy strategy^38^), and done so consistently across each arm.

### 8.2 Sub-group analyses

Given broad eligibility in a pragmatic TB trial, it is valuable to repeat the analysis across key subgroups to understand heterogeneity in the treatment effect. Key subgroups should be few and prespecified to avoid problems with statistical multiplicity^40^, including: participants with bacteriological confirmation of disease, persons with key co-morbidities (such as HIV or diabetes), and priority populations such as children and pregnant women.

## 9 Data collection

The data collection strategy for a pragmatic TB trial should be simple and closely mirror routine practice. A minimal set of variables that directly support the trial’s primary and secondary endpoints should be defined. Scrutiny should be applied to the level of data collection to guard against a somewhat natural research inclination to collect more.

Priority should be given to collecting overarching summary information, focusing on the most informative data, to reduce the burden on clinical staff and support the efficient aggregation of outcomes across multiple sites.

Existing database systems and data entry procedures should be utilized where possible to ensure ease of use and limit on-site training requirements. Each participating institution would retain ownership of its own data, with data-sharing agreements set up to allow for the sharing of data for interim and final statistical analyses. Early engagement with national regulators, national TB programs, ethics committees, and in-country community advisory groups will be important to understand what is appropriate and acceptable in each setting. Consideration for sufficient data quality assurance and quality control procedures are particularly important if data systems are decentralized.

## 10 Trial Monitoring

In a pragmatic trial using registered medications, monitoring should follow a risk-proportionate approach – the scope, frequency, and methods are tailored to the specific trial risks rather than applying a “one size fits all” model. The sponsor must develop a risk assessment and mitigation plan, identify critical data and processes, and define a monitoring plan proportionate to the level of risk (e.g. degree of deviation from usual care).

Following the ICH E6(R3)^41^ quality-by-design framework, the limited factors that are fundamental for the protection of study participants and the reliability of the study results should be identified at the start of the trial. These are called Critical to Quality Factors (CtQFs) and inform the risk-based monitoring strategy. These are also important for pragmatic trials, although they will be different and perhaps fewer than for explanatory trials. The monitoring strategy should include adequate and appropriate quality assurance of microbiology laboratory procedures to reduce variability in diagnosis and monitoring outcomes.

The monitoring burden is reduced by embedding most procedures within routine care: only those processes that diverge from usual practice (e.g. extra sampling, specialized measurements, unblinded data reconciliation) merit direct review or verification beyond standard clinical oversight. All monitoring activities, whether remote or on-site, should be documented and aligned with the monitoring plan. Simplified monitoring must be balanced against trial and data quality, and the CtQFs will help inform an appropriate balance.

A pragmatic TB trial should have oversight from an independent data monitoring committee (IDMC, also known as a Data and Safety Monitoring Board, DSMB) to review safety data at regular intervals, allowing the trial to be stopped early if unacceptable safety signals emerge.

## 11 Other issues

### 11.1 Adaptive elements

Adaptive clinical trial designs are increasingly used in TB trials ^42–49^. These trials have tended to be more explanatory in design and require efficient centralized data management systems and processes for adaptation of randomization during the trial. These are likely not well suited to a pragmatic TB trial.

Areas in which an adaptive design may be appropriate are sample size re-calculation and early stopping for efficacy. Furthermore, if it were possible to quantify the strength of (pragmatic) evidence required to inform a change in international guidelines, methods could be developed to adapt sample size or introduce stopping rules so that the trial is completed as soon as adequate evidence is collected. Bayesian methods are well suited, although more work is needed to explore this approach.

### 11.2 Sub-studies

Sub-studies can play an important role in pragmatic randomized trials by addressing site-or country-specific questions that the main trial is not designed to answer. These could include:

- collecting implementation metrics of the intervention in real-world settings such as acceptability, fidelity and penetration^50^,
- conducting targeted tolerability or safety monitoring in specific subpopulations,
- quality of life and patient preference surveys,
- qualitative semi-structured in-depth interviews,
- studying differential treatment effects in specific populations, and
- collecting additional specimens for pharmacokinetic and minimum inhibitory concentration analyses, or biomarker studies.

In general, sub-studies may be conducted only in a subset of study sites, should be operationally feasible to implement in a real-world setting, and aligned with, and not interfere with, the primary objectives of the parent trial. Sub-studies that require extensive data collection and procedures outside of usual care would likely require additional clinic staff and will impact the pragmatic nature of the trial and this should be avoided.

### 11.3 Variation in arms between sites

Although the 6-month standard of care regimen is routinely used as first-line treatment for DS-TB globally, there is some variation in how it is used including: intermittent dosing in the continuation phase, use of fixed-dose-combination (FDC) tablets, provision of nutritional supplements, provision of financial incentives or enablers, or variation in dosing of individual drugs. In general, delivery of the control arm may vary to align with local standards of care and thus maximize pragmatism.

Variation in the intervention arm between countries and sites (e.g., in dosages or frequency of dosing) will introduce further heterogeneity to the results and may lead to increasing sample size. Some variation may happen in a pragmatic trial, and this can make ultimate policy recommendations more complex if they must account for such variation. The dosing and frequency of the intervention arm should be standardized as much as possible.

## 12 Conclusions

Pragmatic TB trials are needed to inform treatment guidelines that are based on real-world evidence and applicable to the heterogeneous group of people in whom TB treatment is indicated. We have provided some guiding principles to show how these are possible and feasible. No single trial can answer all the important questions pertaining to an intervention regimen; learning and development does not stop at phase 3. Adoption of these principles will improve pragmatic evidence generation to improve the treatment of TB.

## Figures and Tables

**Table 1:** Summary of regimens potentially suitable for a pragmatic TB trial with broad eligibility criteria. The focus is on rifamycin-containing regimens due to the weight of available evidence.

Regimen	Details	Relevant Phase 2 and phase 3 Randomized Clinical Trials
***Regimens potentially suitable for a pragmatic TB trial***		
**4HPMZ**	Four months of isoniazid (H), rifapentine (P), and moxifloxacin (M), supplemented by pyrazinamide (Z) for the first two months.	This regimen was shown to have activity in phase 2 trials^51,52^, and non-inferior efficacy to the control in the Study 31/A5349 phase 3 trial^3^. It is being evaluated in the duration-ranging SPECTRA-TB trial (NCT07595042).
**4HR^h^MZ**	Four months of isoniazid (H), rifampicin (R) at a dose that is higher than usual practice (R^h^), and moxifloxacin (M), supplemented by pyrazinamide (Z) for the first two months.	This regimen represents only a change in rifamycin from the more established 4HPZM regimen and was shown to have activity in a phase 2 trial^44^ and is being evaluated in the ongoing PanACEA STEP2C Phase 2C (NCT05807399) and Phase 3 OptiRiMoxTB (NCT05575518) trials.
**6HR^h^ZE**	Six months of isoniazid (H) and rifampicin (R) at a dose that is higher than usual practice (R^h^) supplemented by pyrazinamide (Z) and ethambutol (E) for the first two months.	Many phase 2 trials have shown increased activity with higher doses of rifampicin^44,53–55^. However, the four- month regimens 4HR^h^ZE (R at 1200mg or 1800mg) were not found to be non-inferior to the control in the Phase 3 RIFASHORT trial. ^56^
***Established standard of care***		
**6HRZE**	Six months of isoniazid (H) and standard-dose rifampicin (R), supplemented by pyrazinamide (Z) and ethambutol (E) for the first two months.	Recommended as first-line therapy in all international guidelines^23^ and used as the control regimen in all phase 3 randomized clinical trials for drug-sensitive TB^57^.

Abbreviation: E – Ethambutol, H – Isoniazid, M – Moxifloxacin, P – Rifapentine, R – Rifampicin, Z – Pyrazinamide, TB - tuberculosis.

**Table 2:** Eligibility considerations for inclusion of priority populations in a pragmatic TB trial.

Priority Population	Considerations
**Children and adolescents**	Adolescents (aged from 12 or 13 years, depending on local definitions) should not be excluded^58^. Pre-adolescent children should also not be excluded, although dosage adjustments will likely be necessary.
**Pregnant and postpartum women**	Every effort should be made to include and retain pregnant and postpartum women in a pragmatic trial^59^. According to a 2025 consensus statement, TB treatment trials should “promote inclusion of pregnant and lactating women (including adolescents) in all TB therapeutic trials as the default approach, unless there is a clear and justifiable rationale not to do so”^60^.
**Persons with extra-pulmonary TB**	There may be severe forms of extrapulmonary TB that might be excluded; however, all persons diagnosed as having extrapulmonary TB and for whom the treating physician would ordinarily prescribe the same treatment as pulmonary TB (6HRZE) should be included in the trial. A bacteriological confirmation of a TB diagnosis may not be possible for many participants with extra-pulmonary TB, and this will have implications for outcomes and analysis (see relevant sections).
**Persons with HIV, Diabetes, or other co-morbidities**	There may be drug-drug interactions between TB drugs under evaluation and other medications that the participants are taking. Such interactions should be managed by adjustment of drugs or dosages of concomitant medications rather than through exclusions, which will limit generalizability and should therefore be avoided.

## Key Points

1. Pragmatic randomized trials are needed to evaluate the effectiveness, safety, and tolerability of TB treatments under routine care conditions to appropriately inform practice guidelines and facilitate uptake.
2. Pragmatic trials are most appropriate for tuberculosis treatments with established efficacy and reassuring safety profiles from prior randomized studies that are feasible for implementation in routine practice.
3. An individually-randomized trial is usually preferred to provide more precise and stronger evidence for guidelines with fewer study participants, although cluster-randomized trials impose less disruption to routine care and so may be more suitable in some settings.
4. Eligibility criteria should be broad, including all people newly diagnosed with TB and being started on TB treatment, irrespective of bacteriological confirmation, and including those with co-morbidities, children, and pregnant women, with a few exclusions.
5. Pragmatic trials should be inegrated within routine clinical services, include flexible adherence support, simplified data collection, and use of standard care pathways wherever medically and ethically appropriate.
6. Sustained cure with disease-free survival should remain the central efficacy outcome for pragmatic tuberculosis trials, while balancing rigorous outcome ascertainment with feasible follow-up approaches suitable for real-world settings.
